# Advancing Cancer Care in Colombia: Results of the First In Situ Implementation of Comprehensive Genomic Profiling

**DOI:** 10.3390/jpm14090975

**Published:** 2024-09-14

**Authors:** Juan Javier López Rivera, Paula Rueda-Gaitán, Laura Camila Rios Pinto, Diego Alejandro Rodríguez Gutiérrez, Natalia Gomez-Lopera, Julian Lamilla, Fabio Andrés Rojas Aguirre, Laura Bernal Vaca, Mario Arturo Isaza-Ruget

**Affiliations:** 1 Laboratorio Clínico Especializado, Clínica Universitaria Colombia, Clínica Colsanitas, Bogotá 111321, Colombia; pauarueda@colsanitas.com (P.R.-G.); lacrios@colsanitas.com (L.C.R.P.); diegoalerodriguez@colsanitas.com (D.A.R.G.); jrlamilla@colsanitas.com (J.L.); 2 Grupo de Genética Médica, Clínica Universitaria Colombia, Clínica Colsanitas, Bogotá 111321, Colombia; 3 Laboratorio Clínico y de Patología, Clínica Colsanitas, Grupo Keralty, Bogotá 111321, Colombia; natalia.gomezl@udea.edu.co (N.G.-L.); fabiarojas@colsanitas.com (F.A.R.A.); misaza@keralty.com (M.A.I.-R.); 4 Servicio de Oncología, Clínica Universitaria Colombia, Clínica Colsanitas, Bogotá 111321, Colombia; laubernal@keralty.co; 5 Grupo de Investigación en Patología Clínica (INPAC), Fundación Universitaria Sanitas, Bogotá 111321, Colombia

**Keywords:** comprehensive genomic profiling, precision medicine, actionable mutations

## Abstract

Background: Comprehensive genomic profiling (CGP) identifies genetic alterations and patterns that are crucial for therapy selection and precise treatment development. In Colombia, limited access to CGP tests underscores the necessity of documenting the prevalence of treatable genetic alterations. This study aimed to describe the somatic genetic profile of specific cancer types in Colombian patients and assess its impact on treatment selection. Methods: A retrospective cohort study was conducted at Clínica Colsanitas S.A. from March 2023 to June 2024. Sequencing was performed on the NextSeq2000 platform with the TruSight Oncology 500 (TSO500) assay, which simultaneously evaluates 523 genes for DNA analysis and 55 for RNA; additionally, analyses were performed with the SOPHiA DDM software. The tumor mutational burden (TMB), microsatellite instability (MSI), and programmed cell death ligand 1 (PDL1) were assessed. Results: Among 111 patients, 103 were evaluated, with gastrointestinal (27.93%), respiratory (13.51%), and central nervous system cancers (10.81%) being the most prevalent. TP53 (37%), KMT2C (28%), and KRAS (21%) were frequent mutations. Actionable findings were detected in 76.7% of cases, notably in digestive (20 patients) and lung cancers (8 patients). MSI was stable at 82.52% and high at 2.91%, whilst TMB was predominantly low (91.26%). Conclusions: The test has facilitated access to targeted therapies, improving clinical outcomes in Colombian patients. This profiling test is expected to increase opportunities for personalized medicine in Colombia.

## 1. Introduction

Precision medicine in oncology involves analyzing the molecular and genetic characteristics of tumors to define personalized treatments. This approach relies on identifying biomarkers that provide a detailed molecular profile of tumor cells, guiding specific therapeutic strategies to improve patient survival and minimize the side effects generated by the use of conventional treatments like chemotherapy [1,2,3]. Precision oncology combines pathology, molecular biology, statistics, bioinformatics, and uses next-generation sequencing (NGS) to explore multiple biomarkers simultaneously, facilitating a more precise definition of therapeutic options [4]. The European Society for Medical Oncology (ESMO) recommends NGS testing for patients with advanced non-squamous non-small-cell lung cancer, prostate cancer, ovarian cancer, and cholangiocarcinoma. Additionally, ESMO suggests analyzing the tumor mutational burden (TMB) in cervical cancers, well- and moderately differentiated neuroendocrine tumors, salivary gland cancers, thyroid cancers, and vulvar cancers [5].

Comprehensive genomic profiling (CGP) looks for all known genomic alterations in a single sample of tumor tissue or blood. This includes base substitutions, copy number alterations, rearrangements, gene fusions, insertions, deletions, and genomic signatures such as the tumor mutational burden (TMB) status, microsatellite instability (MSI), and programmed cell death protein ligand-1 (PD-L1), consolidating these findings into a single assay and report. This comprehensive perspective provided by CGP can reveal targetable alterations that may not typically be associated with specific cancer types [6]. As a result, it offers a more detailed and practical understanding of the genetic landscape of the tumor, which in turn facilitates the development of more precise and personalized therapeutic strategies.

In addition, CGP includes the alterations and biomarkers that can be used to guide treatments with therapies approved by the U.S. Food and Drug Administration (FDA). Also, CGP provides valuable information for the use of other targeted therapies and immunotherapies, including those available through clinical trials. Essential genes for tumor evaluation include EGFR, KRAS, BRAF, and PIK3CA, which play critical roles in the progression of colorectal cancer and in the selection of targeted therapies as anti-EGFR monoclonal antibodies (MoAbs) [2]. Likewise, the evaluation of genes such as ALK, ROS1, RET, and HER2 is crucial in assessing non-small-cell lung cancer (NSCLC), as established in the National Comprehensive Cancer Network (NCCN) guidelines [7].

The identification of additional variants and biomarkers such as gene fusions, TMB, and MSI is essential for defining appropriate therapy in oncology patients. RNA analysis has proven to be a reliable tool for detecting fusions, which are crucial in certain cancers like NSCLC for diagnosis and identifying potential treatments. For example, the EML4-ALK fusion has been identified in at least 2% of patients with NSCLC [8]; this fusion is well studied in the literature and different generations of drugs have been developed to benefit patients with EML4-ALK. On the other hand, TMB and MSI are biomarkers recognized by the FDA and the medical community as important criteria for determining patient eligibility for immunotherapy, regardless of the tumor’s histologic type. Although MSI can be studied through polymerase chain reaction (PCR) or immunohistochemistry (IHC), the simultaneous evaluation of TMB and MSI using next-generation sequencing (NGS) has proven particularly efficient in clinical practice for selecting immunotherapies [9,10].

Alongside pathology studies, the study of PDL1 is conducted. PDL1 is a transmembrane protein considered an inhibitory cofactor of the immune response. Its interaction with PD-1, its natural receptor, modulates the action of T cells against tumor cells expressing PD-L1. This interaction leads to decreased activation, proliferation, cytokine secretion, and the survival of T cells. PD-L1 is expressed in some tumor cells. The PD-L1 and PD-1 interaction is a mechanism by which neoplasms evade the human immune response and are involved in cancer progression. Multiple clinical trials have shown the promising therapeutic effects of immune checkpoint inhibitors targeting PD-1/PD-L1 in solid and hematologic tumors [11,12].

Understanding the molecular profile of certain cancer types is fundamental for defining more effective public health strategies. Access to comprehensive genomic testing in Colombia remains limited, and the prevalence of potentially actionable genetic alterations in this population is not well documented. In Colombia, two studies have highlighted the use of NGS-based tests as a viable option to optimize the selection of personalized therapies and provide relevant prognostic information for cancer patients [13,14]. However, to date, all tumor profiling tests performed on Colombian patients have been conducted abroad. This process can take several weeks, delaying diagnosis and treatment implementation. The local availability of NGS could significantly reduce these times, enabling a faster initiation of targeted therapies. Moreover, it promotes the development of local infrastructure and the training of technical and scientific personnel, enhancing the country’s capacity to conduct high-complexity research and diagnostics. In this context, we present the first in situ implementation of CGP in Colombia, carried out in the specialized laboratory of Clínica Colsanitas. The objective of this study was to conduct a descriptive analysis of the somatic genetic profile of certain types of cancer in Colombian patients and the relationship of genomic findings with available therapeutic strategies.

## 2. Materials and Methods

### 2.1. Design and Participants

We conducted a retrospective cohort study at a single healthcare institution in Colombia, Clinica Colsanitas S.A., from March 2023 to June 2024. The study included 111 patients diagnosed with histologically confirmed malignant neoplasms, including patients with any type of non-hematologic cancer with or without metastasis, patients with unknown primary tumors, and patients with multiple synchronous primary tumors, referred by different oncologists from the main cities of Colombia: Bogota, Ibague, Tunja, Neiva, Bucaramanga, Armenia, Santa Marta, Medellín, Barranquilla, and Cali. The patients ranged in age from 10 to 89 years old, with a mean age of 56.5 years (SD = 16.14), among which 62 (55.86%) were women and 49 (44.14%) were men.

Eight samples (7.20%) could not be processed due to poor material quality. In such cases, a new sample was requested, or, when possible, reprocessing was performed in duplicate. Only one sample was excluded, which had been sent by mistake for a male patient who did not have a cancer diagnosis. In cases where the required minimum DNA concentration was not reached, a new FFPE tissue section was requested from the pathology center; however, in some instances, no additional material was available for reprocessing.

Formalin-fixed, paraffin-embedded (FFPE) tissues were stored in the pathology laboratory within the reference laboratory of Clinica Colsanitas in the city of Bogotá, Colombia, which centralizes all the information on the patients of the national insurer.

### 2.2. Comprehensive Genomic Profiling Test

CGP is based on the TruSight Oncology 500 (TSO500) assay, designed to describe the genomic alterations of a tumor and thus support the definition of treatment in oncology patients. This NGS-based test simultaneously evaluates 523 genes for DNA analysis and 55 for RNA. Additionally, it includes the study of programmed cell death ligand 1 (PD-L1), the most widely used predictive biomarker for guiding patient selection for immunotherapy based on immune checkpoint inhibitors [15].

### 2.3. Covariates

Primary data source was electronic medical records to identify information related to patient demographics and clinical data. These data are stored in secure folders in the cloud and included the following:

- Personal data: Name, age, identification document, city of residence, and sex.
- Medical data: Diagnosis based on histopathologic classification of the tumor, and cancer staging based on TNM Staging System or Stages I–IV. Also, pathology report and information on previous cancer panels and microsatellite instability results.
- Therapy: Details of the response to their first and second line treatments according to the evolution of tumor size.
- Cancer type and sex are nominal categorical variables that were analyzed using absolute and relative frequencies.

### 2.4. DNA and RNA Extraction

The pathologist reviewed and marked unstained FFPE slides to show the affected tumor area and cellularity for future procedures. Tumor tissue was then sliced and placed into a 1.5 mL tube. Nucleic acids were extracted using the DNA and RNA FFPE kit according to the manufacturer’s instructions (AllPrep DNA/RNA FFPE Kit, QIAGEN Inc., Hilden, Germany).

The AllPrep DNA/RNA FFPE procedure started with the removal of paraffin using 100% xylene and 100% ethanol, and then drying the FFPE tissue sections, followed by lysis with proteinase K digestion. The sample was then cooled on ice and centrifuged to obtain an RNA-containing supernatant and a DNA-containing pellet. The RNA was incubated at 80 °C and further processed by binding total RNA, treating with DNase, washing, and eluting. Similarly, the DNA was processed by lysing the pellet with proteinase K digestion, incubated at 90 °C, binding genomic DNA, washing, and eluting. The final products were purified RNA and DNA ready for downstream applications.

DNA and RNA quantification was performed using the Invitrogen Qubit 3.0 fluorometer and the Invitrogen Qubit DNA HS assay kit (Life Technologies, Gaithersburg, MD, USA). Samples were either immediately processed or stored at −80 °C. The quality and integrity of DNA were assessed using real-time PCR (Infinium HD FFPE QC Assay, Illumina Inc., San Diego, CA, USA).

### 2.5. TSO500 Library Preparation and Next-Generation Sequencing (NGS)

The library preparation utilized the Illumina TruSight Oncology 500 DNA/RNA Bundle kit (24 samples) (Illumina, San Diego, CA, USA). Following the extraction and quality control of DNA and RNA from FFPE samples, the genetic material concentration was homogenized, with the manufacturer-recommended initial input concentration of DNA and RNA set at 40 ng.

#### 2.5.1. Library Prep DNA Workflow

The gDNA was fragmented to a size of 90–250 bp per fragment using the Covaris E220 evolution. The 5′ and 3′ strands obtained in the fragmentation process were converted to blunt ends using an A-Tailing end repair mix (ERA1). Then, adapters were ligated to the ends of the gDNA fragments. UMI1 adapters, which ligate only to gDNA fragments, were used. Subsequently, sample purification beads (SPB) were used to purify the gDNA fragments, thereby removing unligated adapters and other unwanted products. Finally, the fragments were amplified using primers that add index sequences for sample multiplexing.

#### 2.5.2. Library Prep RNA Workflow

The purified RNA were denatured and primed with random hexamers. Then, the primed RNA fragments were reverse-transcribed into first-strand cDNA using reverse transcriptase. The RNA template was removed, and double-stranded cDNA was synthesized. SPBs were used to purify the synthesized cDNA. The 5′ and 3′ cDNA strands obtained were converted to blunt ends using an A-Tailing end repair mix (ERA1). Then, adapters were ligated to the ends of the cDNA fragments. SUA1 adapters, which only ligate to cDNA fragments, were used. Subsequently, sample purification beads (SPBs) were used to purify the cDNA fragments, thereby removing unligated adapters and other unwanted products. Finally, the fragments were amplified using primers that add index sequences for sample multiplexing.

#### 2.5.3. Enrichment DNA and RNA Workflow

A group of oligos specific to 523 genes hybridized to DNA libraries and oligos specific to 55 genes hybridized to RNA libraries. Then, streptavidin magnetic beads (SMBs) were used to capture the probes hybridized with the DNA regions of interest from the library. Once these probes were captured, the target regions of the DNA libraries enriched with capture probes were bound a second time. Then, SMBs were used to capture the hybridized capture probes with the DNA regions of interest a second time, and then the enriched libraries were amplified using primers. SPBs were then used to purify the enriched and amplified libraries. The libraries were quantified using fluorometry. The libraries were normalized using bead-based to ensure library uniformity.

Finally, the purified libraries were processed on the NextSeq2000 (Illumina) platform.

### 2.6. Bioinformatic Analysis of Sequencing Data

Sequencing quality parameters such as Phred score (%Q30), clusters passing filters (%PF), percentage of target region covered at different depths 25×, 50×, and 100×, and total reads were analyzed. The bioinformatics analysis of the obtained sequences was conducted using SOPHiA DDM software v4-4.6.2 (https://www.sophiagenetics.com/technology, accessed on 12 June 2024), which includes all secondary pipeline and tertiary analysis tools such as the OncoPortal Plus, powered by the clinical knowledgebase JAX-CKB.

The TMB value was determined by considering all synonymous and non-synonymous variants in coding and high-confidence regions with a depth ≥ 50×. Microsatellite instability (MSI) analysis employs 50 marker loci. All variants that passed the quality filters were used for the comparative analysis.

### 2.7. Variant Analysis and Interpretation

Variants were interpreted according to AMP guidelines [16]. Variants with strong clinical significance were classified as tier I; variants with potential clinical significance were classified as tier II; variants of unknown clinical significance were classified as tier III; and, variants considered benign or probably benign were classified as tier IV. Additionally, variants without clinical actionability but classified as oncogenic and probably oncogenic according to the latest Clingen guidelines are included in the report [17]. Only single nucleotide variants, amplifications, and fusions with tier I and tier II evidence were included in the report. Only FDA-approved drugs were reported. The classification of tumor mutational burden is reported according to Marabelle A. et al. [18], where the TMB-high state is ≥10 mutations per megabase. The actionability associations for the analyzed genes and cancer types were based on NCCN guidelines and supported by databases such as Oncokb and JAX CKB. Before issuance, the findings were discussed with the institution’s medical group of geneticists and oncologists [19].

### 2.8. PD-L1 Expression (Immunohistochemistry) Test

The expression of PD-L1 was analyzed using immunohistochemistry with the anti-PD-L1 antibody clone 22C3 Dako Autostainer (Dako, Carpinteria, CA, USA) on formalin-fixed paraffin-embedded (FFPE) tissue sections previously confirmed to contain tumor cells. Standardized scoring methods were employed: tumor proportion score (TPS) for non-small-cell lung carcinomas (NSCLCs) and combined positive score (CPS) for other solid tumors. PD-L1 positivity was defined as TPS > 1% or CPS ≥ 1 [20].

### 2.9. Ethical Considerations

This study was approved by the Research Ethics Committee of the Fundación Universitaria Sanitas (Protocol 033-22 UNV, Record Number CEIFUS 1108-22). Informed consent was obtained for the use of samples from participating patients in the study. The study was conducted in accordance with local regulations and the Declaration of Helsinki.

### 2.10. Statistical Analysis

We described the molecular profile using descriptive statistics. Categorical variables were presented as frequencies and percentages, while continuous variables were summarized with means and standard deviations.

## 3. Results

### 3.1. Distribution of Cancer

**Figure 1:**
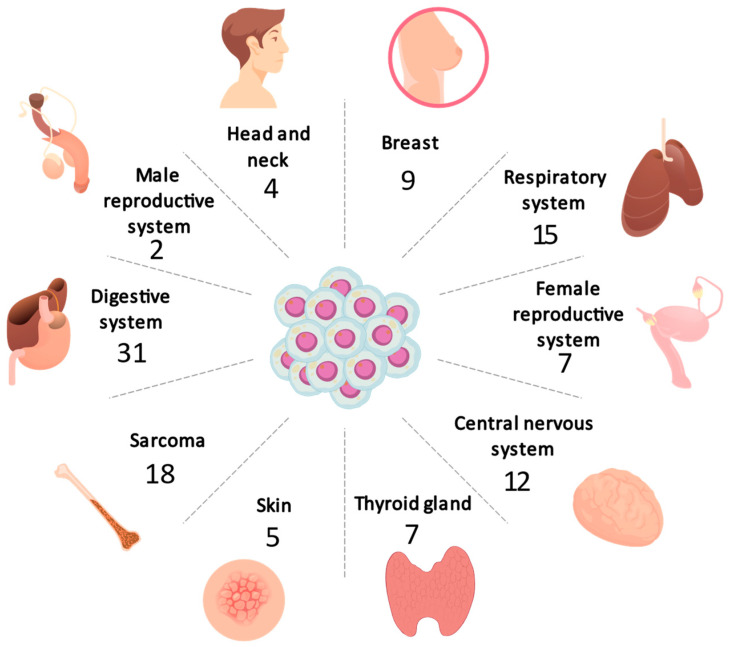
Radial chart demonstrating the distribution of cancer types across various body systems, digit indicates the number of patients affected by system. Note that the digestive system, for example, includes GIST, liver cancer, colorectal cancer, pancreatic cancer, stomach cancer, and bile duct cancer [21].

The most common type of cancer was gastrointestinal (27.93%), followed by respiratory (13.51%), central nervous system (10.81%), sarcoma (11.71%), breast (8.10%), female reproductive system (6.30%), thyroid (6.31%), melanoma (4.50%), head and neck (3.60%), liposarcoma (3.60%), male reproductive system (1.80%), and rare tumors (0.90%). Figure 1 shows the distribution of cancer types according to the system involved and Table 1 indicates the specific type of cancer and the sex of the patients. Three patients presented with synchronous tumors: patient one presented tumors in breast and colon, patient two in prostate and lung, and patient three in breast and biliary tract.

### 3.2. CGP Results

**Figure 2:**
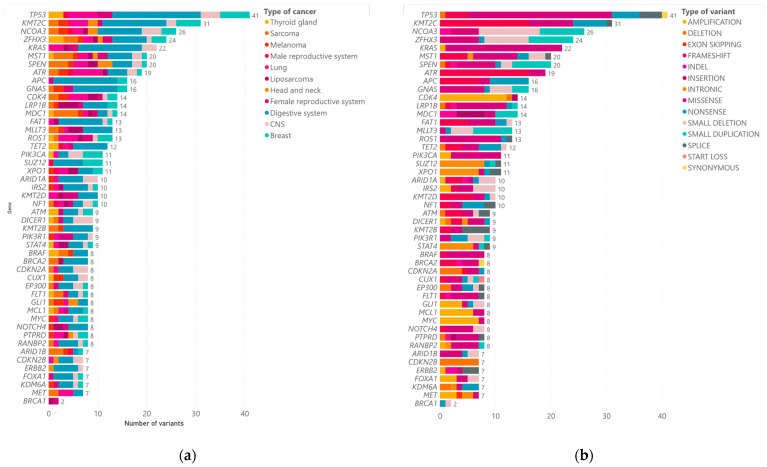
(**a**) The number of genetic variants identified in various cancer types, with each color representing a specific cancer type such as thyroid gland (blue), sarcoma (orange), and melanoma (pink). The horizontal bars represent the total number of variants found for each gene across these cancers, with TP53 and KMT2C having the highest number of variants (41 and 31, respectively). (**b**) A breakdown of the types of genetic variants found, including amplifications, deletions, and missense mutations. Each variant type is color-coded, as described in the legend on the right.

The panel used for tumor genomic profiling includes 523 cancer-relevant genes, of which we obtained at least one finding of clinical significance in 402 genes. A total of 1653 variants were found across these 402 genes. We detected 622 missense variants, 227 frameshift variants, 182 amplifications, 144 small deletions, 140 nonsense variants, 85 deletions, 81 splice variants, 59 small duplications, 48 intronics variants, and 65 other variants (Figure 2). Within this finding, the most frequently mutated genes were *TP53* (37% of cases), *KMT2C* (28%), *ZFHX3* (21%), *NCOA3* (23%), and *KRAS* (21%). Other less frequently mutated genes were *ERBB2*, *FOXA1*, *KDM6A*, *MET*, and *BRCA1*. In various tumor types (including breast, lung, melanoma, prostate, sarcoma, rectum, GIST, and colon), the most common variant observed was *ATR* c.2320dup (p.Ile774fs), which was present in nine patients. Notably, colon tumors exhibited the highest mutation frequency, with 270 variants identified. The average number of readings obtained was 84,425,896.20 with an average mapping percentage of 93.87% (for complete information, please refer to Supplementary Table S1).

### 3.3. Actionable Alterations

**Figure 3:**
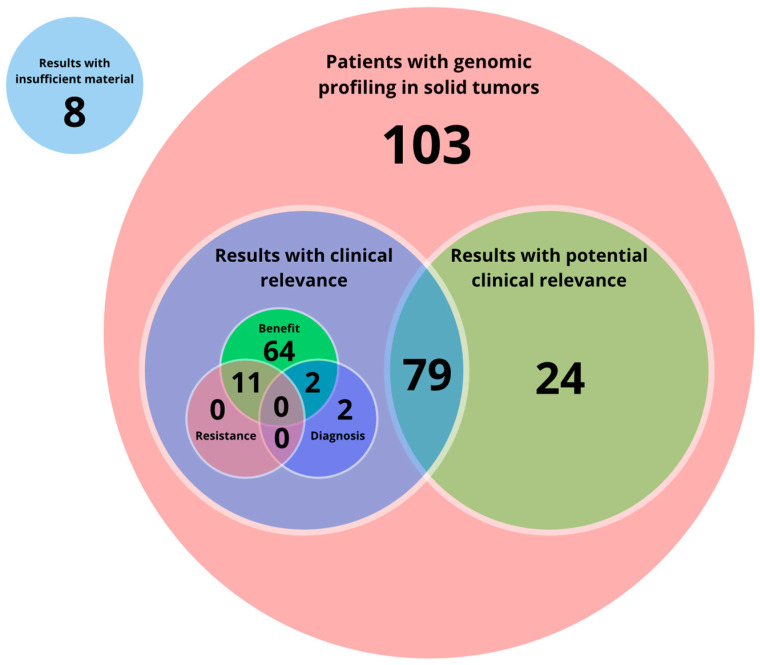
Distribution of clinically relevant and potentially clinically relevant results. Out of 103 patients with solid tumors who underwent genomic profiling, 79 received results with clinical relevance. Among these, 64 showed potential treatment benefits, 11 had resistance-related findings, and 2 had diagnostic significance. In addition, 24 patients received results with potential clinical relevance, while 8 samples were insufficient for analysis.

Genomic profiling was performed in 103 patients, among whom 24 had no actionable findings but other variants of potential clinical relevance. The remaining 76.7% (79 patients) had both actionable and non-actionable findings. Among these, 77 patients had results with at least one molecular profile classified as level IA, IB, IIC, or IID, among which 51 patients (45.94%) had results classified as level IA and/or level IB, while 26 patients (23.42%) had results classified as level IIC and/or level IID, 2 patients had results related to combination therapies and diagnostic evidence, and 11 patients had both benefit and resistance to certain therapies. Other results were diagnosis-related in only two patients (Figure 3) (for complete information, please refer to Supplementary Table S2).

The most relevant findings according to the type of cancer corresponded to the following types:

- Digestive system: This group includes 31 patients, namely 14 men and 17 women with tumors of the rectum, pancreas, liver, intestine, colon and stomach. Out of 31 patients with tumors of the digestive system, positive results (Tier IA/IB) were found in 20 patients (9 men, 11 women), and 1 female patient could not be processed due to poor sample quality. The most frequently mutated genes in this type of cancer were TP53 (51.61%), KMT2C (35.48%), APC (32.25%), NCOA3 (25.80%), and KRAS (29.03%)
- Lung: Out of 14 patients with lung tumors, positive results (Tier IA/IB) were found in 8 patients (3 men, 5 women). The most frequent findings in these positive patients were MET exon 14 (METex14) skipping and EGFR exon 19 deletion. The most frequently mutated genes were ATR (33.3%), EGFR (26.6%), KMT2C (20%), and ROS1 (20%).
- CNS: A total of 12 samples from patients with central nervous system tumors, including glioblastoma, astrocytoma, medulloblastoma, glioma, and meningioma, were analyzed. Out of 12 patients with CNS tumors, positive results (Tier IA/IB) were found in 2 patients (1 men, 1 women), one of which had mutations in the IDH1 gene and the other in the NF1 gene. The most frequent mutations in this type of tumors were in DICER1 (25%) and NCOA3 (25%) genes.
- Sarcoma: Out of 14 patients with sarcoma, positive results (Tier I/II) were found in 2 patients (2 men) with BRAF p.Val600Glu. One patient could not be processed due to poor sample quality. The most frequently mutated genes in this type of cancer were SPEN (28.57%), NUTM1 (21.42%), MST1 (21.42%), and ZFHX3 (21.42%).
- Breast: Out of nine patients with breast tumors, positive results (Tier IA/IB) were found in five patients (five women). The most frequently mutated genes were TP53 (55.55%), ZFHX3 (44.4%), SPEN (55.5%), PIK3CA (33.3%), and SUZ12 (44.4%).
- Female reproductive system: Out seven patients with female reproductive system tumors, positive results (Tier IA/IB) were found in three patients (42.85%). Half of the patients with ovarian tumors had mutations in TP53 and the only patient with endometrial cancer had an oncogenic variant in BRCA1. The most frequently mutated genes in this type of cancer were TP53 and KMT2D (42.85%).
- Thyroid gland: Out of seven patients with thyroid gland tumors, positive results (Tier IA/IB) were found in four patients (two women, two men), whilst three patients could not be processed due to poor sample quality. Two patients had BRAF p.Val600Glu variant findings. ZFHX3 (42.85%) was the most frequently mutated gene.
- Melanoma: Out of five patients with thyroid gland tumors, positive results (Tier IA/IB) were found in two patients (one man, one woman) with BRAF p.Val600Glu.
- Head and neck: Out of four patients with head and neck tumors, none were found to be positive (Level I/II). However, the PAX3-FOXO fusion was found in a 17-year-old patient with an adenoid cystic carcinoma of the trachea.
- Liposarcoma: Out of five patients with liposarcoma, positive results (Tier IA/IB) were found in two patients (two women). The most frequently mutated gene was LRP1B (75%).
- Male reproductive system: Positive results (Level IA/IB) were found in two of two patients (100%) evaluated with tumors in the male reproductive system.
- Rare tumors: In this group, there is a male patient with a diagnosis of PEComa. The recommended treatment for this finding consisted of mTOR inhibitors, such as sirolimus, temsirolimus, and everolimus.

### 3.4. MSI and TMB

**Figure 4:**
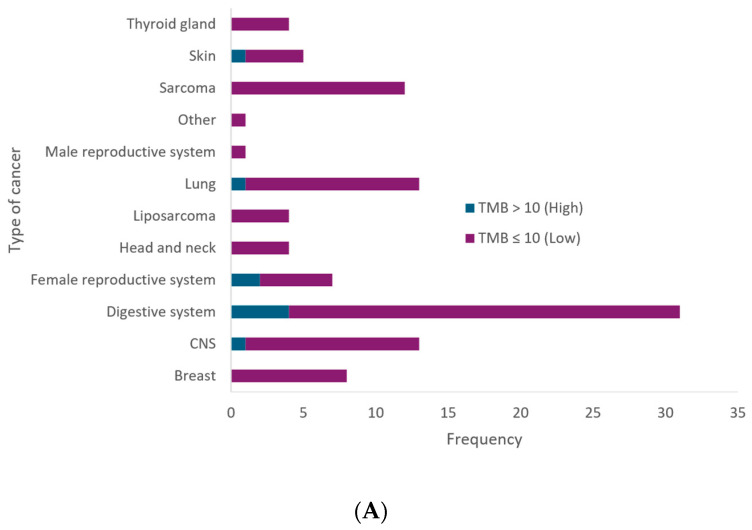

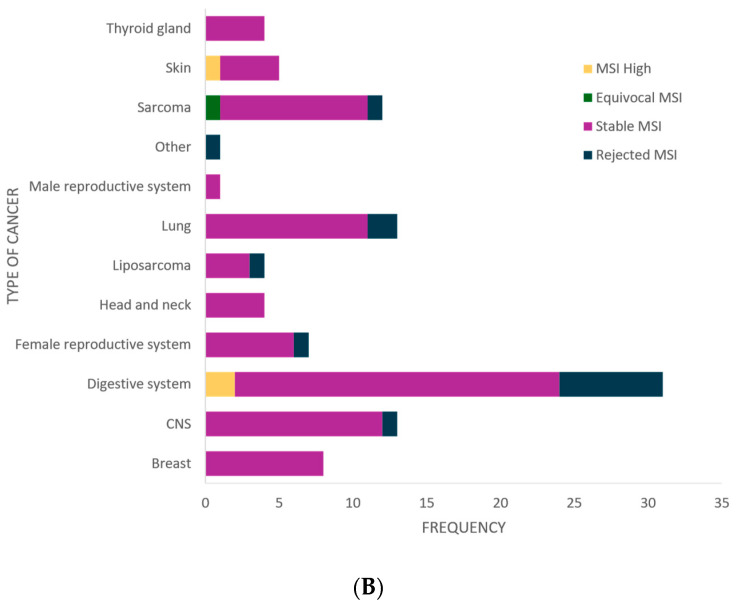
TMB and MSI across different cancer types. (**A**) Bar chart displaying the frequency of cancer types with high (TMB > 10) and low (TMB ≤ 10) tumor mutational burden. (**B**) Bar chart illustrating the frequency of MSI status across different cancer types. The chart categorizes MSI status into high, equivocal, stable, and rejected MSI.

Additionally, our findings revealed that 82.52% of cases exhibited microsatellite stability (MSS), 2.91% showed high MSI (referred as MSI), and 13.59% were rejected due to low sample quality. Within cancers of the digestive system, two cases of high MSI were identified. Similarly, a single case of high MSI was observed in skin cancer. MSI was actionable in one female patient with sigmoid colon cancer (Figure 4A).

The analysis of tumor mutational burden (TMB) across different types of cancer reveals that the majority of cases, 91.26%, present a low TMB (≤10 mutations per megabase), indicating a lower number of mutations in the tumor DNA (Figure 4B). High TMB (>10 mutations per megabase) was observed in 8.74% of cases. The cancer types with the highest incidence of high TMB include the digestive system, with four cases, while one case of high TMB was identified in cancers of the central nervous system, female reproductive system, and skin. Additionally, a high tumor mutational burden was found in a patient with primary CNS sarcoma (TMB: 52.31), a patient with sigmoid colon cancer (TMB: 140.3) and a patient with non-small-cell lung cancer (NSCLC) (TMB: 32.3). In addition, actionability was found in the type of tumor presented by five patients (9.61%), three females, and two males with sigmoid colon cancer, primary sarcoma of the CNS, moderately differentiated squamous cell carcinoma of the cervix, melanoma and thyroid cancer, respectively.

### 3.5. PD-L1 Expression (Immunohistochemistry)

**Figure 5:**
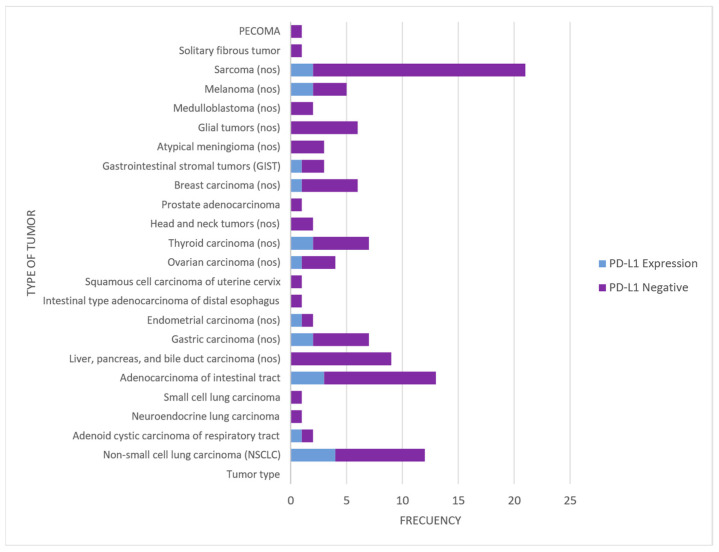
PD-L1 expression across different tumor types. Bar chart visualizing the PD-L1 expression by tumor type. The chart contrasts the number of tumors with PD-L1 expression (lilac bars) versus those that are PD-L1 negative (purple bars).

The expression of PD-L1 by tumor type is summarized in Figure 5 and Table 2:

## 4. Discussion

The tumor genomic profile is a crucial tool in oncology for defining patient-specific treatments by understanding the tumor’s genetic makeup and offering personalized medications. This approach is gradually replacing conventional, non-selective standard therapies due to the development of drugs targeting specific tumor mutations. The proven clinical benefits of certain targeted therapies, the growing number of actionable biomarkers, and the decreasing cost of CGP have significantly increased the demand for this type of analysis. CGP has been reported to be cost-effective compared to conventional genomic tests from the perspective of the Colombian healthcare system [22]. Given these advantages, this study describes our experience with the clinical use of CGP to assess the somatic genetic profile in specific types of cancer among Colombian patients and its impact on treatment selection.

This study found that gastrointestinal cancer was the most common, accounting for 27.93% of cases, followed by respiratory system cancers and central nervous system cancers. This highlights the significant impact of gastrointestinal cancers in this population, which is consistent with global data showing a high incidence of these types of cancer worldwide [23,24]. Gastrointestinal cancers, including stomach, colon, rectum, liver, and pancreas tumors, make up a substantial portion of the global cancer burden, representing 26% of all cancer cases and 35% of cancer-related deaths [25].

During this study, 92.79% of the samples were found suitable for genomic testing, a percentage higher than what has been reported in other studies [26]. Moreover, 73% of the patients had an informative or actionable genomic alteration. Out of these, 64 had molecular profiles that could benefit from specific therapies, including those classified as IA, IB, IIC, and IID. This finding aligns with a study of 250 hematologic tumors, which stated that around 75% of patients possess a genomic alteration that can be targeted with an approved drug [27]. Similarly, a study in the Colombian population indicated that, out of 153 patients with enough tissue samples, 84% had actionable genomic alterations that could be treated with FDA-approved drugs specific to the patient’s tumor type (46.4%) or with treatments used for a different tumor type (37.6%) [13]. These results emphasize the crucial role of genomic profiling in identifying potential therapeutic targets and integrating these alterations into standard care for precise therapeutic stratification, thereby advancing precision oncology and enhancing patient outcomes.

The high frequency of variants in genes such as *TP53*, *KMT2C*, *NCOA3*, *ZFHX3*, and *KRAS* underscores their crucial role in oncogenesis and their potential as therapeutic targets. *TP53* mutations, prevalent in over half of all human cancers, often lead to loss of tumor-suppressive functions and gain of oncogenic properties [27,28]. KMT2C mutations, frequently co-occurring with TP53 mutations, are linked to specific cancer subtypes and may predict responses to immune checkpoint inhibitors [29]. NCOA3, significant in melanoma susceptibility, is emerging as a therapeutic target due to its frequent amplification in cancers [30]. These findings highlight the importance of these genes in cancer progression and their potential for targeted therapies, offering a promising direction for personalized cancer treatment and improved patient outcomes. Additionally, the ATR c.2320dup (p.Ile774fs) variant found in multiple tumor types suggests its critical role in the pathogenesis of various cancers, offering a potential target for future therapies.

Among the most relevant actionable findings, we found a simultaneous amplification of *PDL1*, *PDL2*, and JAK2 (9p24.1) in a patient with a sigmoid colon adenocarcinoma, which is a rare genomic event reported in 0.7% of 187 tumor samples of >100 tumor types, including the presence of this marker in 0. 18% of colorectal cancer samples [31]. The identification of amplifications in PDL1 is important because this subset of tumors appears to have a high probability of responding to an immune checkpoint blockade (ICI) (anti-PD-1 agents: Nivolumab, Pembrolizumab; anti-CTLA-4 agents: Ipilimumab; anti-PD-L1 agents: Atezolizumab, Avelumab) with a response rate of 66.7% among 9/13 patients with *PDL1* amplification treated with ICI [32,33].

Interestingly, a rare variant in *PIK3C2G* c.4460G>C (p.*1487Sext*4) was identified in a female patient with two synchronous cancers (breast and biliary tract cancer), which according to the literature and the COSMIC database, has only been identified in a single other biliary tract sample in a patient with biliary tract adenocarcinoma [34]. Although this variant is not actionable and has not been defined as a determinant in patients with this diagnosis, sharing this type of finding could support the scientific community in the identification of new critical genes in each type of cancer, and in the future, use these as biomarkers or therapeutic targets.

Regarding biomarkers, the deficiency in MMR leading to MSI has been widely described in various types of human cancer, most commonly in colorectal, endometrial, and gastric adenocarcinomas [35,36]. In our study, cases of high MSI in cancers of the digestive system and skin suggest a subgroup of patients who could significantly benefit from immunotherapy. Notably, one patient with stage IV colon cancer and high MSI is benefiting from first-line immunotherapy with pembrolizumab.

In our population, 10% of colorectal cancer tumors were found to have microsatellite instability. This finding contrasts with previous reports on the prevalence of MSI in tumors reported in other ethnic groups, where an average MSI of 12% in African Americans and 14% in Caucasians has been reported. Additionally, the previously reported MSI in colorectal cancer tumors is 17% [33].

It has been demonstrated that CGP can accurately assess TMB compared to whole-exome sequencing [37]. In the analyzed patient sample, 96% showed a low tumor mutational burden (TMB). This finding may be associated with a lower potential response to certain immunotherapies that rely on a high mutational burden to be effective [38]. Despite the prevalence of low TMB, the identification of 8.74% of cases with high TMB underscores the heterogeneity of tumors and their potential response to immunotherapies. This variability is consistent with other prospective studies, such as one that described 235 patients who underwent integrated NGS profiling and also found that the median TMB was low [37], similar to a sample of 170 Colombian patients [13].

In patients with gastric cancer, studies demonstrate that those with colon and small intestine cancer have a higher TMB within the group of gastrointestinal tumors, which is consistent with the results obtained in our study [39]. In breast cancer patients, the mean TMB was 2.83 muts/Mb, a value similar to that reported in studies with large cohorts of breast cancer patients (2.63 muts/Mb). Among breast cancer subtypes, the median TMB was higher in patients with triple-negative breast cancer (4.60 muts/Mb) compared to hormone receptor-positive patients (3.10 muts/Mb) [40].

In addition, the immunophenotypic findings of PD-L1 expression in tumor cells and intratumoral inflammatory cells have proven relevant as a method of evaluation to establish an independent prognostic factor in several solid and hematologic tumors and even in some as a favorable indicator of immune therapy response. However, it is not without limitations, including the subjectivity and adherence to test interpretation criteria, the use of multiple PD-L1 clones (22C3, SP142, SP263, 28.8), the existence of several protocols for the interpretation of the test (TPS, IC, CPS), and the standardization of cut-off points only for some solid tumors such as triple-negative breast cancer, NSCLC, cervical carcinoma, squamous cell carcinoma of the head and neck, and gastroesophageal junction carcinoma [41,42,43,44]. While protocols supported by proven data are already available for many tumors, dedicated studies and clinical trials focusing on the harmonization of the topic in other still only partially explored fields are surely advisable [12,20].

While this study provides valuable insights into the treatable alterations identified through GCP in Colombia, several limitations should be acknowledged. Firstly, the sample size is constrained. Given that Colombia is a developing country and considering that the data presented here were collected over one year, access to this test for patients remains limited within the country. Although it is now possible to conduct these tests locally, they are still often outsourced to external laboratories. Consequently, the sample size in this study was restricted.

Additionally, in this study, 14 patients were identified in whom the processed tissue did not match the tissue of primary tumor origin. Accuracy in tumor tissue sampling is crucial for genetic profiling and cancer characterization. Intratumor heterogeneity, i.e., variability among cancer cells within the same tumor, can lead to significant differences in genetic and molecular test results. This underscores the importance of obtaining representative samples from the primary tumor. Samples obtained from locations other than the tumor origin may not accurately reflect the biology of the primary cancer and thus could lead to the misinterpretation of tumor aggressiveness, treatment response potential, and prognosis. Therefore, it is essential to standardize sampling procedures and ensure that they are collected as accurately and consistently as possible to improve the reliability of oncologic studies [45].

Despite these limitations, the study exhibits notable strengths. It provides the first comprehensive insights into treatable genetic alterations identified through CGP in Colombia across more than 500 genes, contributing valuable data to the field. Also, this is the first study in Colombia that performed GCP in situ and admitting patients from different regions of the country. Overall, the study’s meticulous approach and thorough documentation of genomic data represent significant strengths in advancing personalized medicine options for cancer patients in Colombia.

## 5. Conclusions

Implementing CGP in Colombia is a significant advancement in oncology, particularly in a developing country. This study emphasizes how CGP enhances the knowledge and skills of laboratory personnel and physicians, making it easier to access targeted therapies for cancer patients. The results show that CGP provides a reliable molecular profile, which supports the use of precision medicine in clinical practice.

The successful application of this technology in Colombia is a milestone in expanding personalized medicine. CGP not only significantly improves clinical outcomes and reduces mortality in cancer patients, but also demonstrates the feasibility of conducting high-complexity genomic testing locally. This reduces the dependence on foreign laboratories and shortens the time between diagnosis and treatment. This local capability accelerates access to precision medicine, encourages infrastructure development, and promotes technical training within the country, setting a crucial precedent for precision oncology in the region.

The findings reveal a high prevalence of actionable genetic alterations, particularly in gastrointestinal, lung, and breast cancers, with key mutations such as TP53, KMT2C, and KRAS. These alterations directly impact the selection of targeted therapies, enhancing the precision of treatment options. The study also emphasizes the importance of biomarkers like high TMB and MSI in specific cancer subtypes, such as colorectal and melanoma, which benefit from immunotherapy. This highlights the need to integrate CGP into daily clinical practice for therapeutic decisions based on the molecular characteristics of each tumor.

In conclusion, this study provides solid evidence for the integration of CGP into the standard care of cancer patients in Colombia. This integration facilitates personalized treatment strategies that improve clinical outcomes and advance precision oncology in the region.

## Figures and Tables

**Table 1:** Distribution of cancer types by gender and specific organ.

Type of Cancer	Male		Female		Total	
	*n*	%	*n*	%	*n*	%
Liver	2	1.8%	1	0.9%	3	2.7%
Colorectal	5	4.5%	5	4.5%	10	9.0%
Pancreas	3	2.7%	1	0.9%	4	3.6%
Gastric	3	2.7%	7	6.3%	10	9.0%
GIST	1	0.9%	1	0.9%	2	1.8%
Bile duct	0	0.0%	2	1.8%	2	1.8%
Lung	7	6.3%	8	7.2%	15	13.5%
Astrocytoma	0	0.0%	2	1.8%	2	1.8%
Glioblastoma	2	1.8%	0	0.0%	2	1.8%
Glioma	2	1.8%	0	0.0%	2	1.8%
Meningioma	1	0.9%	2	1.8%	3	2.7%
Medulloblastoma	1	0.9%	1	0.9%	2	1.8%
Primary CNS sarcoma	0	0.0%	1	0.9%	1	0.9%
Sarcoma	9	8.1%	5	4.5%	14	12.6%
Breast	0	0.0%	9	8.1%	9	8.1%
Cervical	0	0.0%	1	0.9%	1	0.9%
Ovarian	0	0.0%	4	3.6%	4	3.6%
Endometrial	0	0.0%	1	0.9%	1	0.9%
Uterus	0	0.0%	1	0.9%	1	0.9%
Thyroid gland	4	3.6%	3	2.7%	7	6.3%
Melanoma	2	1.8%	3	2.7%	5	4.5%
Nose	1	0.9%	1	0.9%	2	1.8%
Salivary gland	1	0.9%	0	0.0%	1	0.9%
Trachea	0	0.0%	1	0.9%	1	0.9%
Liposarcoma	2	1.8%	2	1.8%	4	3.6%
Prostate	2	1.8%	0	0.0%	2	1.8%
PEComa	1	0.9%	0	0.0%		
Total	49		62		111	

**Table 2:** Table summarizing the frequency and PD-L1 expression status across various tumor types. The table lists the number of cases for each tumor type and indicates the percentage of tumors that are positive for PD-L1 expression.

Tumor Type	Frequency	PD-L1 Expression
Non-small-cell lung carcinoma (NSCLC)	12	4 (33%)
Adenoid cystic carcinoma of respiratory tract	2	1 (50%)
Neuroendocrine lung carcinoma	1	0
Small-cell lung carcinoma	1	0
Adenocarcinoma of intestinal tract	13	3 (23%)
Liver, pancreas, and bile duct carcinoma (nos)	9	0
Gastric carcinoma (nos)	7	2 (29%)
Endometrial carcinoma (nos)	2	1 (50%)
Intestinal type adenocarcinoma of distal esophagus	1	0
Squamous cell carcinoma of uterine cervix	1	0
Ovarian carcinoma (nos)	4	1 (25%)
Thyroid carcinoma (nos)	7	2 (29%)
Head and neck tumors (nos)	2	0
Prostate adenocarcinoma	1	0
Breast carcinoma (nos)	6	1 (17%)
Gastrointestinal stromal tumors (GIST)	3	1 (33%)
Atypical meningioma (nos)	3	0
Glial tumors (nos)	6	0
Medulloblastoma (nos)	2	0
Melanoma (nos)	5	2 (40%)
Sarcoma (nos)	21	2 (10%)
Solitary fibrous tumor	1	0
PECOMA	1	0

## Data Availability

The data that support the findings of this study are available on request from the corresponding author. The data are not publicly available due to privacy and ethical restrictions.

## Supplementary Materials

The following supporting information can be downloaded at: https://www.mdpi.com/article/10.3390/jpm14090975/s1.
